# Layered Double Hydroxides-Loaded Sorafenib Inhibit Hepatic Stellate Cells Proliferation and Activation *In Vitro* and Reduce Fibrosis *In Vivo*

**DOI:** 10.3389/fbioe.2022.873971

**Published:** 2022-05-27

**Authors:** Wei Peng, Shiwen Zhang, Wei Zhou, Xinchen Zhao, Kexue Wang, Chengxu Yue, Xinyu Wei, Siyan Pang, Wei Dong, Sulian Chen, Changjie Chen, Qingling Yang, Wenrui Wang

**Affiliations:** ^1^ Anhui Province Key Laboratory of Translational Cancer Research, Department of Biotechnology, Bengbu Medical College, Anhui, China; ^2^ Department of Biochemistry, School of Laboratory Medicine, Bengbu Medical College, Anhui, China; ^3^ The Third Department of Hepatic Surgery, Eastern Hepatobiliary Surgery Hospital, Second Military Medical University, Shanghai, China

**Keywords:** layered double hydroxides, sorafenib, liver fibrosis, hepatic stellate cells, TGF-β1/Smad

## Abstract

A core feature of liver fibrosis is the activation of hepatic stellate cells (HSCs), which are transformed into myofibroblasts and lead to the accumulation of extracellular matrix (ECM) proteins. In this study, we combined *in vitro* cellular efficacy with *in vivo* antifibrosis performance to evaluate the outcome of sorafenib (SRF) loaded layered double hydroxide (LDH) nanocomposite (LDH-SRF) on HSCs. The cellular uptake test has revealed that sorafenib encapsulated LDH nanoparticles were efficiently internalized by the HSC-T6 cells, synergistically inducing apoptosis of hepatic stellate cells. Moreover, the apoptosis rate and the migration inhibition rate induced by LDHs-SRF were 2.5 and 1.7 times that of SRF. Western Blot showed that the TGF-β1/Smad/EMT and AKT signaling pathway was significantly inhibited in HSC-T6 cells treated with LDHs-SRF. For the *in vivo* experiment, LDHs-SRF were administered to rat models of CCl_4_-induced liver fibrosis. H&E, masson and sirius red staining showed that LDHs-SRF could significantly reduce inflammatory infiltrate and collagen fiber deposition and immunohistochemical results found that LDHs-SRF treatment significantly inhibited the protein expressions of α-SMA in the liver, these results suggesting that LDHs-SRF exhibited better anti-fibrotic effect than SRF alone and significantly inhibited the proliferation and activation of rat hepatic stellate cells and collagen fiber synthesis.

## Introduction

Liver fibrosis is a dynamic process that results from liver cell damage caused by various causes, including toxins, alcohol, hepatitis virus, non-alcoholic steatopathies, and autoimmune hepatitis (Poilil Surendran et al., 2017). When the liver is damaged, it triggers a physiological repair response to maintain normal tissue function. However, this repair response often leads to liver fibrosis, which instead leads to loss of cell function, damage to tissue structure, and even fatal organ failure or liver cancer if timely treatment is not provided. Currently, no standard treatment exists for liver fibrosis. Elimination of the etiological factor is considered the most effective therapy. Studies show that natural compounds have a lot of advantages such as abundant reserves, wide sources, and relative safety. Developing natural drugs with anti-fibrosis effects has broad prospects and great significance. However, herbal medicines such as curcumin (Hanafy, 2021) and silybin (Ou et al., 2018) are insoluble in water, resulting in low bioavailability in the body. Other drugs, such as pirfenidone (Gan et al., 2021), have toxic side effects that limit their effectiveness in the treatment of liver fibrosis. With the development of nano drug delivery system, more and more nano carriers have been designed to deliver drugs, which increases the high efficiency of drug targeting and greatly reduces the adverse reactions of drugs (Panyam and Labhasetwar, 2003; Hanafy et al., 2019; Hanafy et al., 2020). The nano-drug delivery system is a new and safe drug delivery system which are widely used in the field of liver fibrosis. The precise delivery of liver fibrosis drugs using nanocarriers provides a new idea for the treatment of liver fibrosis.

The activation and proliferation of hepatic stellate cells (HSCs) is considered as a key event in the pathogenesis of hepatic fibrosis. After liver injury, HSCs are activated by inflammatory mediators and then differentiate into myofibroblasts, which initiate the liver tissue remodeling process through the secretion of extracellular matrix (ECM) proteins and matrix metalloproteinases induced by HSCs (Tsuchida and Friedman, 2017; Dewidar et al., 2019).

Sorafenib (SRF) is a multi-target receptor tyrosine kinase inhibitor. As the first oral drug approved by the FDA for treating hepatocellular carcinoma, it is predominantly used in clinical practice (Ma et al., 2017). Recent studies have found that SRF can effectively inhibit the proliferation and promote the apoptosis of HSCs, which provides new horizons for the treatment of liver fibrosis (Wang et al., 2010; Ganten et al., 2017; Su et al., 2018). However, it is associated with significant side effects such as fatigue, diarrhea, high blood pressure, nausea, and skin toxicity, severely reducing the patient’s quality of life and limiting the therapeutic effect. In addition, SRF is difficult to dissolve in water, the bioavailability in the body is extremely low, seriously impacting its efficacy (Kim et al., 2017; Abdelgalil et al., 2019). The mechanisms underlying the efficient and stable activity of SRF on target organs/target cells remain unclear, warranting further studies to improve current anti-liver fibrosis drug formulations.

Layered double hydroxides (LDHs), also known as natural hydrotalcite, are a new type of inorganic nanomaterials with positively charged layers and an interlayer structure of exchangeable anions. It has been widely used as a nano-delivery vehicle for drugs or biologically active substances (Chaillot et al., 2021). LDHs have many advantages as nano-delivery carriers, including good biocompatibility and biodegradability, as well as drug sustained and pH-sensitive release characteristics. Moreover, LDHs enable the loading of small molecule inhibitors, oligonucleotides, plasmids, antigen polypeptides and other biological drugs and maintain the pharmacological properties of the drugs. In addition, LDHs are injectable and suitable for intravenous administration. Last but not least, the artificial synthesis of LDHs is relatively cheap and eco-friendly, suggesting that LDHs have great commercial value in the field of drug delivery (Zhu et al., 2016; da Costa Fernandes et al., 2019; Gutierrez-Gutierrez et al., 2020). The current study aimed to load sorafenib into LDHs, hoping to develop an actively targeted nanomedicine for liver-fibrosis therapy and improve the bioavailability and efficiency of sorafenib. Physicochemical characterization was performed. We further assessed the therapeutic effect of LDHs-SRF on activated HSCs *in vitro* and the anti-fibrotic effect of LDHs-SRF on a rat model of CCl_4_-induced hepatic fibrosis *in vivo*.

## Materials and Methods

### Materials, Cell Line, and Animal Model

MgCl_2_·6H_2_O (97%), AlCl_3_·6H_2_O (97%), NaOH (97%), HCl (36%–38%), and SRF were purchased from Aladdin Reagent Co., Ltd. (Shanghai, China). DMEM (High sugar) and Fetal bovine serum (FBS) were obtained from ThermoFisher Biochemical Products Co., Ltd. (Beijing, China). Dimethyl sulfoxide (DMSO), cell count kit-8 (CCK-8), Annexin V–FITC/PI, bicinchoninic acid (BCA) protein assay, and SDS-PAGE were purchased from Beyoyime (Nanjing, China). All antibodies were purchased from ABclonal (Wuhan, China).

HSC-T6 cells were obtained from COBIOER Biosciences Co., Ltd. (Nanjing, China). Cells were maintained in complete DMEM supplemented with 10% fetal calf serum, 1% penicillin, and 1% streptomycin in 75 ml flasks at 37°C and 5% CO_2_.

Sprague-Dawley (SD) male rats were purchased from Jinan Pengyue Experimental Animal Breeding Co., Ltd. (Jinan, China). All animal studies were conducted in accordance with accepted standards of animal care under a protocol approved by the Bengbu Medical College Animal Ethics Committee.

### Preparation of Layered Double Hydroxides and Layered Double Hydroxides-Loaded Sorafenib

LDHs were synthesized by hydrothermal and co-precipitation techniques. 40 ml of a metal mixed salt solution was prepared, consisting of 609.9 mg of magnesium chloride (MgCl_2_·6H_2_O) and 241.4 mg of aluminum chloride (AlCl_3_·6H_2_O). 1 M NaOH solution was added to adjust the pH value under nitrogen atmosphere, at 60°C and pH conditions of 10 ± 0.01. The mixture was then incubated in a water bath for 1 h. After centrifugation, the supernatant was discarded to obtain the initial precipitation products of LDHs, which were resuspended in 40 ml deionized water, in a hydrothermal reactor at 100°C for 16 h, and then allowed to cool. After centrifugation at 7,600 rpm for 10 min, the cells were washed twice with deionized water, and 10 ml of the LDHs suspension obtained was resuspended in deionized water and freeze-dried in a lyophilizer for later use. (The deionized water used in the synthesis process has removed CO_2_).

LDHs-SRF were also synthesized by hydrothermal and co-precipitation techniques. A 10 ml metal salt solution containing MgCl_2_·6H_2_O (3 mmol) and AlCl_3_ 6H_2_O (1 mmol) was first obtained, then the SRF mixture (200 mg SRF was dissolved in 0.1 ml DMSO and then transferred to 40 ml 1 M NaOH for ultrasonic mixing) was added. The mixture was incubated in a water bath under nitrogen conditions at 80°C and stirred for 2 h. After centrifugation, the supernatant was discarded. This procedure was repeated twice. The obtained precipitate was resuspended with 40 ml of CO_2_ deionized water. The hydrothermal reaction was conducted at 100°C for 16 h in an autoclave. A uniformly dispersed milky liquid was obtained after cooling. The supernatant was discarded by centrifugation, washed twice with deionized water, resuspended in 10 ml of deionized water, and stored for later use in a freeze dryer.

### Characterization

A drop of the diluted solutions of LDHs and LDHs-SRF was placed onto a carbon-coated copper TEM grid (150 mesh, Ted Pella Inc., Rodding, CA). The samples were imaged using a JEM-1230 transmission electron microscope (JEOL Ltd., Japan) and visualized at 120 kV under a microscope. The particle size and the polydispersity index at 25°C were determined by photon correlation spectroscopy (Zetasizer Nano ZS, Malvern Instruments, Malvern, United Kingdom). Zeta potential values were estimated based on electrophoretic mobility using the same equipment at 25°C. The colloid stability of LDHs-SRF in PBS and serum medium was investigated. Fourier transform infrared spectroscopy (FT-IR) was used to characterize LDHs and LDHs-SRF nano-formulations. The measurements were carried out in the range between 400 and 4,000 cm^−1^ on a Bruker Vector 22 FTIR spectrophotometer (Bruker AXS, Inc., Billerica, MA, United tates). X-ray powder diffraction (XRD) patterns were recorded from 5° to 80° using Cu Kα radiation (*λ* = 1.54060 Å, 40 kV, 40 mA, step of 0.0330°) on a Rigaku Miniflex diffractometer (Rigaku Corporation, Tokyo, Japan).

### Determination of Drug Loading of Layered Double Hydroxides-Loaded Sorafenib Preparation

Ultraviolet-visible absorption spectroscopy was used to determine the content of SRF in LDHs-SRF preparations. The SRF solution was prepared in methanol to achieve concentrations of 0.5, 1, 2, 4, 8, 16 μg/ml, and their corresponding absorbance at 265 nm was measured. Fitting the working curve of SRF in methanol solution, the linear formula of absorbance and concentration of SRF in methanol was obtained. 10 mg of dry powder of LDHs-SRF was dissolved into added to 3 ml methanol. Then methanol was added to make a final volume of 10 ml, and the mixture was mixed by ultrasonication. Subsequently, the concentration of SRF was determined by monitoring the absorbance at *λ*max = 265 nm with UV–vis spectroscopy. The following formula was used:

$$\text{D}\text{r}\text{u}\text{g}\;\text{l}\text{o}\text{a}\text{d}\text{i}\text{n}\text{g}\;\%\;=\frac{\text{c}\text{o}\text{n}\text{c}\text{e}\text{n}\text{t}\text{r}\text{a}\text{t}\text{i}\text{o}\text{n}\;\text{o}\text{f}\;\text{S}\text{R}\text{F}\;\text{i}\text{n}\;\text{L}\text{D}\text{H}\text{s}-\text{S}\text{R}\text{F}}{\text{ concentration of feeding sorafenib}}\times 100\%$$

### *In Vitro* Cell Uptake Assay

2 mg LDHs-SRF and 2 mg FITC were dissolved in 1 ml pure water and mixed by ultrasound for 30 min. Then, the mixture was placed at 4°C overnight. The next day, the mixture was centrifuged at 3,000 rpm/min for 5 min, three times, until the supernatant was clarified to get LDHs-SRF (stained with FITC) and freeze-dried for later use.

5 × 10^3^ HSC-T6 cells were inoculated into 24-well plate (14 mm, Solarbio, China) and treated with 10 μM LDHs-SRF (stained with FITC) at different time points (1, 4, 8, 12, 24 h), respectively. The cell plate was washed with PBS twice, and the cell plate was fixed. The results were observed with confocal laser scanning microscope (Olympus FV-1200MPE, Japan).

For flow cytometer analysis, 1 × 10^6^ HSC-T6 cells were inoculated into 6-well plate and cultured for 12 h. Then, treated with 10 μM LDHs-SRF (stained with FITC) at different time points (1, 4, 8, 12, 24 h). The cells were collected and washed twice with PBS, then the cells were resuspended with 2% paraformaldehyde for flow cytometry.

For TEM observation, briefly, the cells treated with LDHS-SRF for 24 h were collected and fixed with 2.5% glutaraldehyde. After being washed twice by PBS, the cells were fixed with 1% precooled osmium tetroxide for 1 h. Then the cells were dehydrated in gradient ethanol (30%, 50%, 70%, 80%, 90%, 95%, 100%) and incubated with acetone and embedding agent (1:1) at 37°C for 4 h. Ultrathin slicing machine 70 nm ultrathin slice and transfer to 150 mesh copper film mesh. According to the standard staining method, 2% uranium acetate was first stained, and then 2.6% lead citrate was counterstained. After drying, the images were observed under HT7800 TEM (HITACHI Ltd., Japan).

### Cell Proliferation Assay

The CCK-8 assay was used to detect the effects of SRF, LDHs and LDHs-SRF on the viability of hepatic stellate cells. HSC-T6 was uniformly seeded in 96-well plates at a density of 5 × 10^3^ cells per well and grown in 100 μl DMEM medium for 24 h. Following the attachment, the cells were incubated in a fresh medium (100 μl/well) containing SRF (5, 10, 15 μM), LDHs-SRF (5, 10, 15 μM of SRF), and LDHs, respectively. According to the CCK-8 kit reagent instructions, the absorbance value was measured at a wavelength of 450 nm to detect cell viability.

### Cell Apoptosis

HSC-T6 cell apoptosis detection: The Annexin V-FITC/PI method was used to detect the effect of the LDHs-SRF nano preparation on HSC-T6 cell apoptosis. HSC-T6 was inoculated into a six-well plate at a density of 4 × 10^5^ per well and cultured for 24 h. Except for the blank control group, after washing HSC-T6 with PBS, the cells in the experimental group were incubated in a medium containing SRF, LDHs, and LDHs-SRF, at a concentration equivalent to 10 μM SRF at 37°C and placed in a 5% CO_2_ constant temperature incubator for 24 h. The cells were then digested with 0.25% trypsin without EDTA to collect the cells. After washing twice with pre-cooled PBS, 500 μl of binding buffer diluent was added. Next, 5 μl of FITC-labeled phospholipid-binding protein V (Annexin V-FITC was added) and 5 μl PI were added, and the mixture was well stirred at room temperature in the darkness for 15 min. Within 1 h, the flow cytometer (FACS) was used for observation and detection.

### Cell Cycle

The propidium iodide method was used to detect the effect of LDHs-SRF nano preparation on the HSC-T6 cell cycle. The HSC-T6 cells were inoculated into a six-well plate at a density of 4 × 10^5^ per well and cultured for 24 h. Unlike the blank control group, cells in the experimental group were treated with a medium containing SRF, LDHs, and LDHs-SRF, at a concentration of 10 μM SRF. The culture was continued at 37°C and 5% CO_2_ for 24 h. Thereafter, the cells were washed with pre-cooled PBS, digested with 0.25% trypsin (without EDTA) to collect the cells. Then, cells were washed again with pre-cooled PBS and fixed in 70% ethanol for 24 h. After the fixation, the cell pellets were collected by centrifugation. After washing the cells with pre-cooled PBS, RNase (100 μg/ml) and PI (50 μg/ml) were added, and the mixture was incubated at 37°C for 30 min in the dark. The cell cycle was detected by flow cytometry. Modfit 5.0 was used to analyze the experimental results.

### Migration Test

Cell Scratch Test: HSC-T6 cells were inoculated into a six-well plate at a density of 4 × 10^5^ cells/well with a total volume of 2 ml per well and pre-cultured for 24 h at 37°C, 5% CO_2_ and saturated humidity to ensure that the cell fusion rate was at least 95%. A 10 μl pipette tip was used to make a scratch repeatedly. After the supernatant was discarded, the suspended cells with phosphate buffer saline (PBS) were washed, and 2 ml suspensions containing different concentrations of SRF, LDHs, and LDHs-SRF were added (Based on the concentration of SRF, 10 μM). The cells were cultured in a DMEM medium (with 1% FBS added) for 24 h. In the control group, cells were treated with a blank medium (containing 1% FBS). Three replicate wells were used in each group. Pictures were taken and recorded with an inverted microscope at 0 and 24 h.

Transwell Test: HSC-T6 cells were incubated in different medium containing sorafenib, LDHs or LDHs-SRF (concentration 10 μM) for 24 h. Cells from different treatment groups were inoculated with 5 × 10^3^ cells into the upper chamber of transwell (medium without FBS) and the lower chamber of transwell was inoculated with complete medium containing 15% FBS, and cultured for 24 h. The cells that did not pass through the transwell were washed with PBS, fixed with 4% paraformaldehyde for 20 min, and stained with crystal violet dye for 40 min. Wash off excess dye and let dry. Pictures were taken and recorded with an inverted microscope.

### Western Blot Assay

HSC-T6 cells in the logarithmic growth phase were inoculated into a six-well plate at a density of 4 × 10^5^ cells/well with 2 ml per well at 37°C. The mixture was placed in a 5% CO_2_ incubator for 24 h. The original medium was discarded, and 2 ml of medium containing different concentrations of SRF, LDHs, and LDHs-SRF were added. After 24 h of treatment, the cells of each group were collected, and the protein extract was prepared at the ratio of 1 ml cell lysate to 10 μl phenylmethylsulfonyl fluoride (PMSF) to extract the protein. The protein levels were quantified by the bicinchoninic acid (BCA) method. After protein samples were separated by SDS-PAGE gel electrophoresis, they were transferred to a polyvinylidene fluoride (PVDF) membrane, which was blocked with skimmed milk, and incubated with primary and secondary antibodies. The protein expression of collagen I, alpha-smooth muscle actin (α-SMA), TGF-β1, Smad7, AKT, p-AKT, Vimentin, Snail, E-cadherin and N-cadherin in each group were finally visualized on a gel imager.

### Rat Liver Fibrosis Model

32 Sprague-Dawley (SD) male rats were randomly divided into 4 groups (8 in each group): Control group, CCl_4_+LDHs group, CCl_4_+SRF group, and CCl_4_+LDHs-SRF group. We adopted the model of liver fibrosis induced by CCl_4_. Olive oil and CCl_4_ were fully mixed at 4:1 ratio to obtain 20% CCl_4_ solution, which was injected intraperitoneally at a dose of 2 ml/kg twice a week for 8 weeks to establish the model of liver fibrosis. The dose of CCl_4_ was based on previous studies (Mu et al., 2018; Zhou et al., 2021), Rats in the control group were administered with the same dose of olive oil. In the first week after the successful modeling, the intervention was carried out by tail vein administration. The dose of CCl_4_+SRF group was 10 mg/kg. According to the loading amount of LDHs-SRF, the dose of CCl_4_+ LDH_S_-SRF group was 50 mg/kg, and the dose of CCl_4_+LDHs group was 40 mg/kg, three times a week, until the end of the fifth week. The rats were then sacrificed, and the corresponding specimens were taken.

### H&E, Masson and Sirius Red Staining

To evaluate the histopathological changes in liver tissue, H&E staining was used to identify inflammatory infiltration and cell necrosis, Furthermore, masson and sirius red staining were used to reflect the degree of collagen deposition in liver tissue (Rittie, 2017; Bansod et al., 2020).

1) H&E staining: Sections were deparaffinized, rehydrated, and stained with hematoxylin for 10 minutes. The tissues were then washed with running water for 10 min, followed by counterstaining with eosin for 5 min. After differentiation, the sections were dehydrated in 80%, 95%, and 100% ethanol, followed by clearing in xylene. Neutral gum sealing was performed, and the tissues were permanently mounted. Pictures were taken with an advanced upright microscope.
2) Masson staining: The dewaxed and hydrated sections were stained with hematoxylin dye for 10 min, fully washed with water, and differentiated with acidic ethanol. Masson de Ponceau S acid reddish staining solution was dyed for 8–10 min, followed by 0.2% glacial acetic acid and phospho-molybdenum pickling for 1 min, and dyeing with aniline blue for 1–2 min. Wash with 0.2% glacial acetic acid for 1 min, dehydrate transparently and seal with neutral gum. The results were observed under an ordinary optical microscope.
3) Sirius red staining: The dewaxed and hydrated sections were stained with sirius red staining solution for 1 h and then rinsed with running water to remove the staining solution. The cells were stained with Mayer hematoxylin staining solution for 8–10 min and rinsed with running water for 10 min. After conventional dehydration, neutral gum sealing was performed. The results were observed under an ordinary optical microscope.

### Immunohistochemical Staining

In order to evaluate the expression of α-SMA in liver tissues, immunohistochemical staining was used to locate, qualitatively and relatively quantitatively the labeled antigens in the tissues by using the specific binding of antigens and antibodies and the principle of chemical coloration. In simple terms, paraffin-embedded liver tissue sections were dewaxed to hydration, then the antigen was repaired with citric acid buffer, then the endogenous H_2_O_2_ enzyme in the tissues was blocked with 3% H_2_O_2_, and then sealed with 3% BSA. The primary antibody was incubated for 4°C overnight, and the second antibody of goat and rabbit was incubated for 30 min the next day, and stained with color developing agent diaminobenzidine (DAB). After washing, redyeing, transparent, and sealing, the images were observed and photographed under a microscope and analyzed.

### Statistical Analysis

Experiments were performed in triplicate and data are expressed as means ± SD. The one-way ANOVA statistical method was carried out to evaluate the significance of the experimental data using SPSS software. A value of 0.05 was selected as the significance level and the data are indicated with (*) for *p* < 0.05, (**) for *p* < 0.01, and (***) for *p* < 0.001.

## Results

### Characterization of Layered Double Hydroxides and Layered Double Hydroxides-Loaded Sorafenib

**FIGURE 1:**
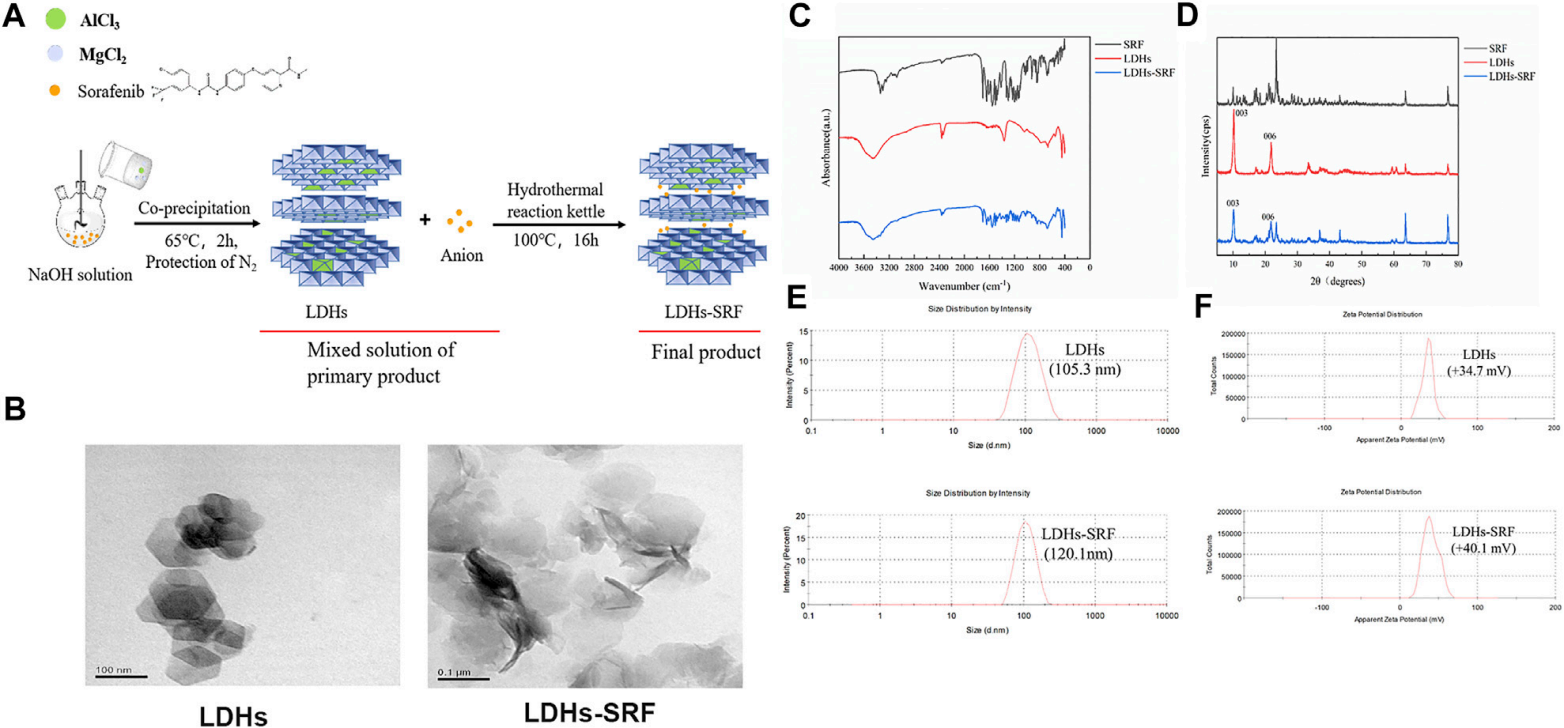
The Characterization of LDHs and LDHs-SRF. **(A)** Schematic diagram of synthesis path of LDHs-SRF; **(B)** TEM image of LDHs and LDHs-SRF; The scale bar in TEM images: 100 nm. **(C)** FT-IR spectra of SRF, LDHs and LDHs-SRF; **(D)** XRD patterns of SRF, LDHs and LDHs-SRF; **(E)** The size distribution of LDHs and LDHs-SRF, PDI represent polymer dispersity index; **(F)** Zeta potential of LDHs and LDHs-SRF in PBS.

The synthesis of LDHs/LDHs-SRF by the hydrothermal and co-precipitation technique is shown in Figure 1A. Our results showed that LDHs and LDHs-SRF were successfully prepared. Furthermore, TEM image showed polygonal sheet without SRF and with SRF the structure was changed into rods (Figure 1B). The FT-IR spectra of LDHs and LDHs-SRF nano preparations are shown in Figure 1C. No new absorption peaks were observed within the wavenumber range of 4,000–400 cm^−1^, indicating that no new chemical bonds were formed between SRF and LDHs, which were instead bound by physical adsorption. As shown in Figure 1D, the XRD data demonstrated the existence of characteristic diffraction peaks (003) and (006), indicating the integrity of the LDHs-SRF crystal structure. As shown in Figure 1E, the average particle size of LDHs and LDHs-SRF particles was 105.3 nm and 120.1 nm, respectively. The particle size distribution of both particles was relatively dense, and the variation in particle size distribution was in accordance with the volume change trend before and after the loading of LDHs drugs. As seen in Figure 1F, the average zeta potential of LDHs and LDHs-SRF was +34.7 mV and +40.1 mV, respectively. There was no significant change in zeta potential before and after LDHs were loaded with SRF. However, as shown in Supplementary Figure S1, when LDHs-SRF suspension was mixed with serum-medium, the average particle size was increased from 120.1 nm (LDH in PBS) to 517.2 nm (LDHs in serum medium), ascribed to rapid protein adsorption on the LDH surface and formation of LDH aggregates. The zeta potentials were decreased from +40.1 mV of LDHs-SRF in PBS to −13.9 mV in serum-medium (Supplementary Figure S1). The significant decrease of the zeta potential is mainly attributed to negatively charged serum proteins adsorption on the surface of LDH nanoparticles.

### Drug Loading Analysis

**FIGURE 2:**
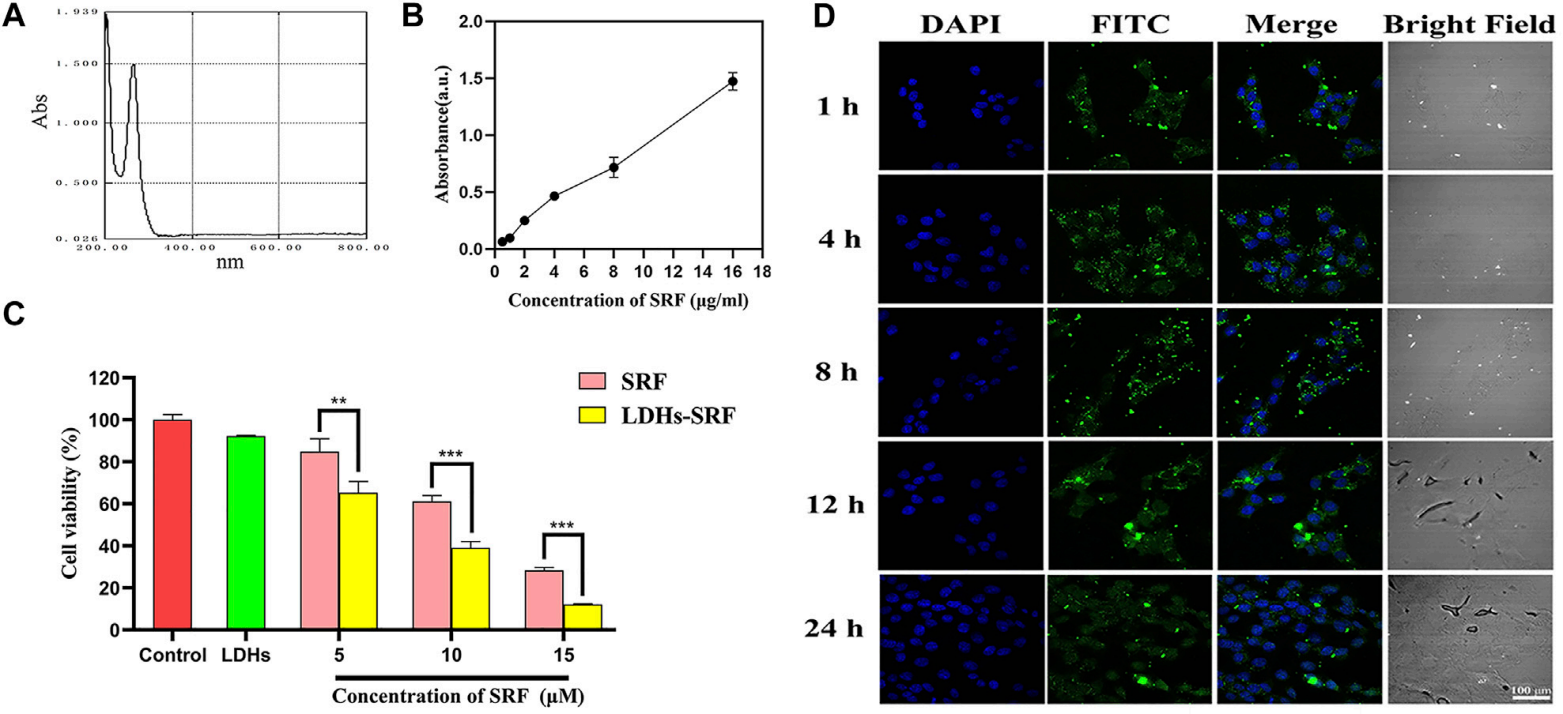
Cell proliferation ability and cellular uptake of LDHs-SRF in HSC-T6 cells. **(A)** Ultraviolet full wavelength scanning curve of SRF; **(B)** Correction curve of SRF in methanol; **(C)** CCK8 analysis of the cell viability of HSC-T6 treated with sorafenib, LDH, and LDHs-SRF after 24 h treatment. Data are expressed as mean ± SD (*n* = 3) and use one-way ANOVA with Tukey’s multiple comparison test, **p < 0.01, ***p < 0.001; **(D)** Confocal images of LDHs-SRF (stained with FITC) and nucleus (DAPI) at different time points. (Scale bar = 100 μm).

The UV full wavelength scanning curve of SRF was obtained, and the maximum absorption peak of SRF was observed at 265 nm (Figure 2A). The working curve of SRF in methanol was then drawn (Figure 2B). The following linear regression equation was obtained: Abs = 0.1076c − 0.0052, (*R* ^2^ = 0.9875, c represents the concentration of SRF in the sample to be measured). The sorafenib loading ratio in LDHs-SRF nanocomposite was about 25%.

### Enhanced Cellular Uptake and Cytotoxicity of Layered Double Hydroxides-Loaded Sorafenib Treatment in Hepatic Stellate Cells-T6 Cells

In order to verify whether LDHs-SRF was successfully delivered to target cells, we examined the internalization of LDHs-SRF(FITC) by HSC-T6 cells in the experiments by the CLSM. As shown in Figure 2D, the cellular uptake appeared at 1 h and remained stable and long lasting at 4, 8, and 24 h. FACS analysis (Supplementary Figure S2) also proved that the cellular uptake appeared at 1 h of 91.75%. As shown in Supplementary Figure S3, LDHs-SRF was observed inside the cells and mainly located in the cytoplasm after 24 h incubation. This means that LDH can serve as a desirable nanocarrier to deliver drugs into the cytosol to induce the cytotoxicity. The CCK-8 assay assessed the effect of free sorafenib and LDHs-SRF on the proliferation inhibition of HSC-T6 cells at different concentration at 24 h. There was significant difference between sorafenib and LDHs-SRF treated groups for 24 h, while as concentration increased from 5 to 15 µM. LDHs-SRF treated HSC-T6 showed a dose dependent cytotoxicity. The inhibition rates of SRF and LDHs-SRF with 10 μM sorafenib on HSC-T6 cells were 39% and 61%, respectively (Figure 2C).

### Effect of Layered Double Hydroxides-Loaded Sorafenib on Cell Apoptosis and Cycle

**FIGURE 3:**
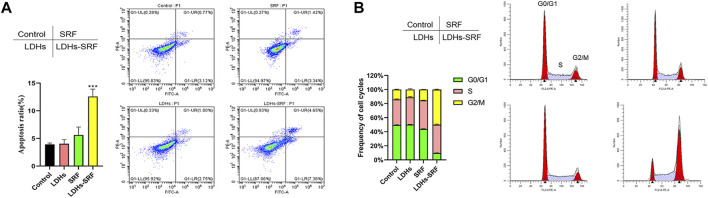
Effects of SRF and LDHs-SRF on Cell Apoptosis and Cell cycle. **(A)** FACS detection of apoptotic cells of HSC-T6 treated with LDHs, sorafenib and LDHs-SRF for 24 h. **(B)** FACS detection of cell cycle analysis of HSC-T6 treated with LDHs, sorafenib and LDHs-SRF for 24 h.

As shown in Figure 3A, flow cytometry showed that the apoptosis induction rates of HSC-T6 treated with 10 μM sorafenib and LDHs-SRF were 4.76% and 12.01%, respectively at 24 h. Cell cycle analysis was conducted to explore the effect of SRF and LDHs-SRF on HSC-T6 cells. As shown in Figure 3B, no significant change in G2/M phase HSC-T6 cells was observed after treatment with 10 μM SRF. In contrast, the G0/G1 phase HSC-T6 cells were significantly inhibited after 10 μM LDHs-SRF treatment, with a significant number of the cells arrested in the G2/M phase.

### Layered Double Hydroxides-Loaded Sorafenib Inhibited the Migration of Hepatic Stellate Cells-T6 Cells *In Vitro*

Cell migration refers to the movement of cells after receiving a migration signal or sensing a concentration gradient of certain substances (e.g., serum). Common methods for detecting cell migration include scratch assay and transwell assay. The former compares the migration rate of cells by measuring the width of the scratch area before and after the scratch treatment, while the latter compares the migration rate of cells by counting the number of cells passing through the transwell. Cell migration is a common mode of movement in living cells, and the migration ability of normal cells will be inhibited after injury.

**FIGURE 4:**
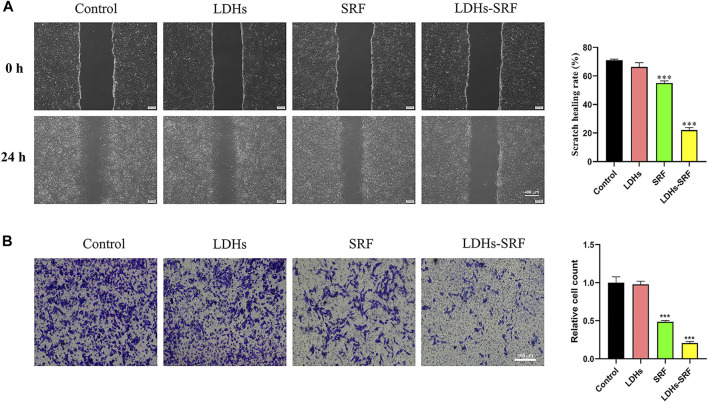
Effect of 10 μM SRF and LDHs-SRF on HSC-T6 cells migration capacity for 24 h. **(A)** Cell scratch test, the scale bar: 400 μm; **(B)** Transwell test, the scale bar: 100 μm. Data represent mean ± SD (*n* = 3) and use one-way ANOVA with Tukey’s multiple comparison test, ***p < 0.001 vs. Control.

Compared with resting HSCs, activated cells exhibit stronger proliferative ability, accompanied by drastic changes in stellate morphology and imbalances in extracellular matrix homeostasis. Interestingly, it has been shown that activated HSCs present strong migration ability and participate in the migration of white blood cells to the damaged liver site. Accordingly, we evaluated the effect of LDHs-SRF on the migration ability of HSC-T6 cells. Through the scratch and transwell migration assays, we found that the cell migration ability in both SRF and LDHs-SRF groups was inhibited compared with the control group. During the scratch assay, we found that the rate of cell migration in the SRF and LDHs-SRF groups were about 55% and 22%, respectively (Figure 4A). During the transwell migration assay, we consistently found that the migration rates were about 48% and 20%, respectively (Figure 4B). Compared with SRF treatment alone, HSC-T6 cell migration was significantly inhibited in the LDHs-SRF group.

### Layered Double Hydroxides-Loaded Sorafenib Downregulates the Expression of α-SMA and Collagen I in Hepatic Stellate Cells-T6 Cells Through the TGF-β1/Smad Mediated EMT Signaling Pathway and AKT Signaling Pathway

**FIGURE 5:**
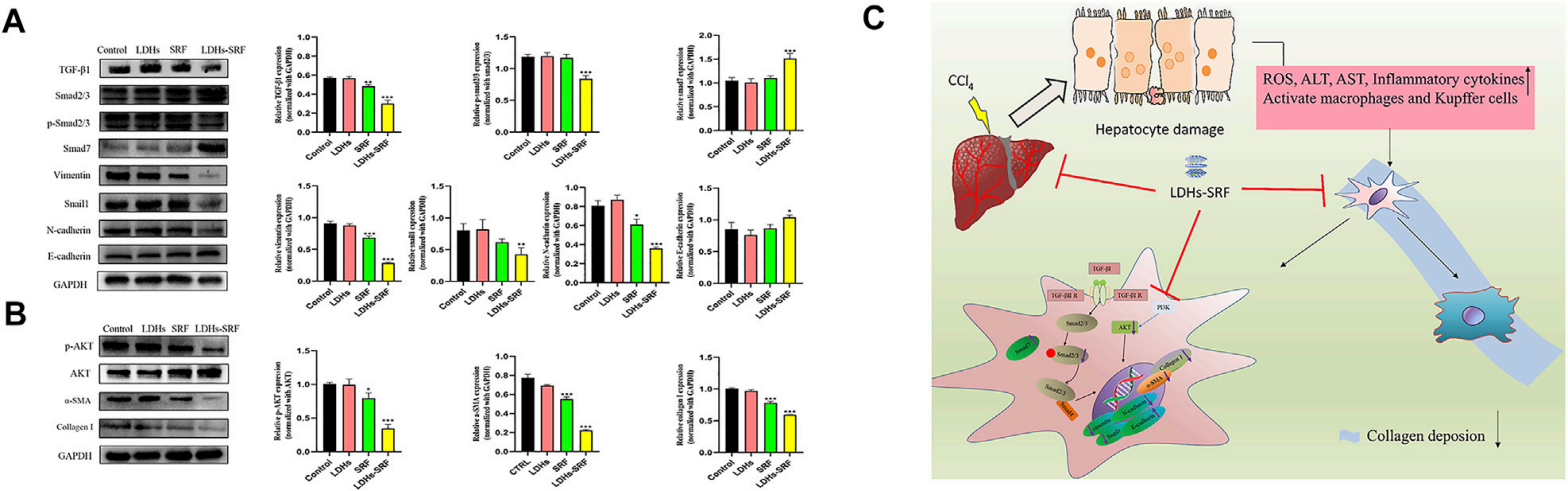
Effects of LDHs-SRF on HSC-T6 through TGF-β1/Smad EMT and AKT signaling pathway. **(A)** TGF-β1/Smad/EMT signaling pathways, showing representative western blot and quantitative analysis graphs for TGF-β1, Smad2/3, p-Smad2/3, Vimentin, Snail1, N-cadherin, E-cadherin; **(B)** AKT signaling pathway, showing representative immunoblots and quantitative analysis graphs for p-AKT, AKT, α-SMA, Collagen I; **(C)** Schematic diagram of the molecular mechanism of LDHs-SRF in HSC-T6 cells. Data represent mean ± SD (*n* = 3) and use one-way ANOVA with Tukey’s multiple comparison test, *p < 0.05, **p < 0.01, ***p < 0.001 vs. Control.

Members of the TGF-β family can be divided into three subtypes, namely TGF-β1, TGF-β2, and TGF-β3, among which TGF-β1 has been documented to be the main signaling pathway during the activation of hepatic stellate cells. The effects of LDHs-SRF on the TGF-β1/Smad/EMT signaling pathway in HSC-T6 cells were detected by Westernblot (Figure 5A). The results showed that TGF-β1 and Smad7 (the inhibitor of TGF-β1) were downregulated and upregulated in both SRF and LDHs-SRF groups, respectively, compared to the control group. Based on this finding, it can be concluded that LDHs-SRF exerted a more significant suppressive effect on TGF-β1 signal transduction. The expression levels of markers vimentin, snail1, N-cadherin and E-cadherin, proteins associated with the EMT signaling pathway regulated by TGF-β1, were detected by Westernblot (Figure 5A). Compared with the free drug group, the expression of E-cadherin was significantly increased, while the expression of vimentin, snail1 and N-cadherin was significantly downregulated in the LDHs-SRF group. Moreover, p-AKT expression in the PI3K/AKT signaling pathway was significantly inhibited in the LDHs-SRF group. LDHs-SRF induced the downregulation of activation markers α-SMA and ECM Collagen I in HSC-T6 cells through the TGF-β1/Smad induced EMT signaling and AKT signaling pathways (Figure 5B).

### Layered Double Hydroxides-Loaded Sorafenib Improve the Antifibrotic Effects of Sorafenib in a Rat Model of CCl_4_-Induced Liver Fibrosis *In Vivo*

CCl_4_ is a drug with selective hepatotoxicity, which induces necrosis of liver cells around central veins in hepatic lobules and then causes fibrosis hyperplasia, mainly in the hepatic sinusoidal space. CCl_4_-induced liver fibrosis model is the earliest and most widely used model of liver fibrosis in laboratory, and has been widely reported in liver fibrosis studies (Dong et al., 2016; Tsuchida et al., 2018; Wu et al., 2020).

**FIGURE 6:**
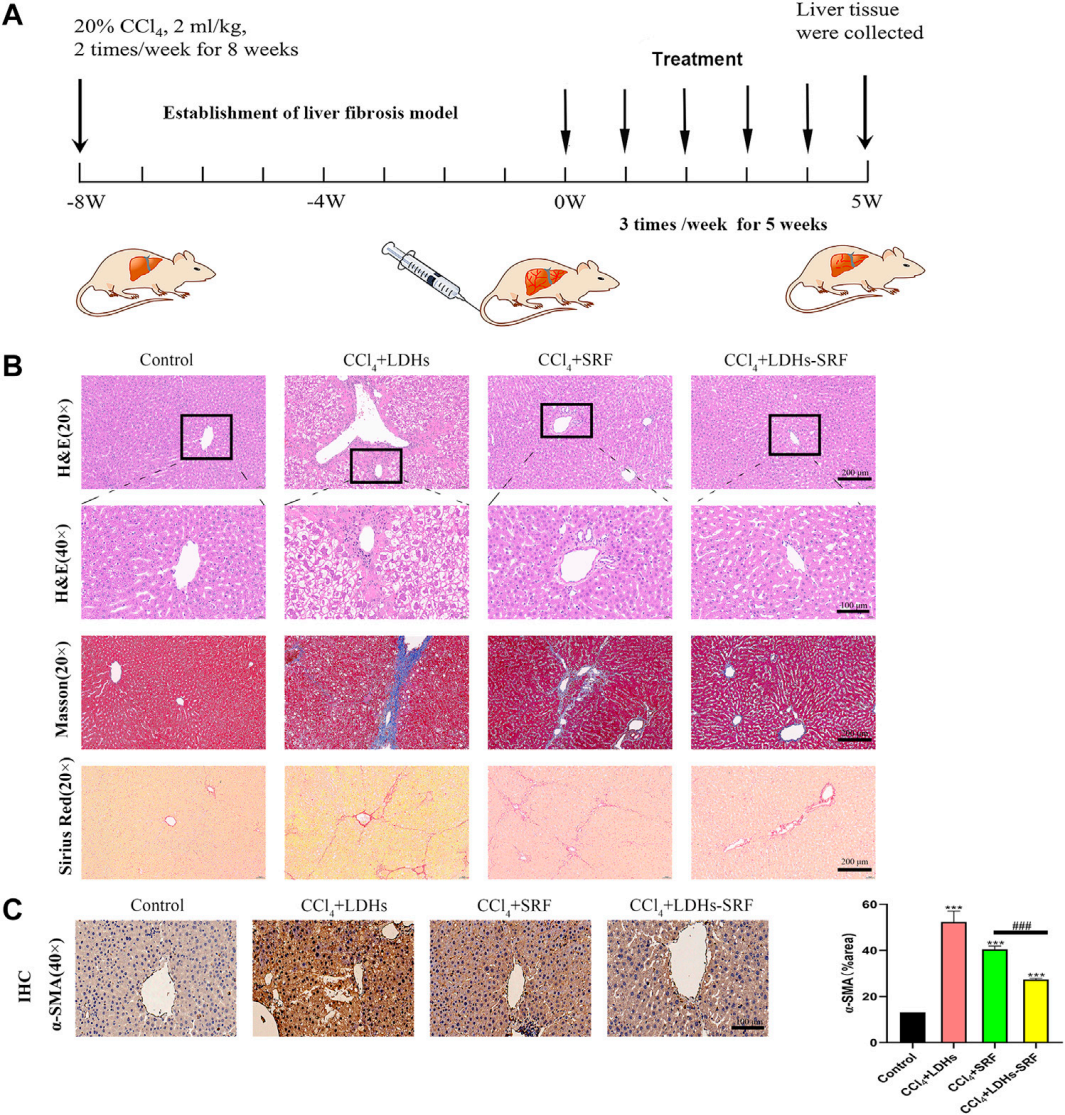
The therapeutic effect of LDHs-SRF on CCl_4_-induced liver fibrosis model in SD rats. **(A)** Flow chart of CCl_4_-induced liver fibrosis model in SD rat; **(B)** Representative of H&E, masson and sirius red staining of liver tissue section; **(C)** Representative of immunohistochemical staining and quantitative analysis graphs of α-SMA in liver tissue section. Data represent mean ± SD (*n* = 3) and use one-way ANOVA with Tukey’s multiple comparison test, ***p < 0.001 vs. Control, ^###^ *p* < 0.001.

In order to explore the effect of LDHs-SRF on CCl_4_-induced liver fibrosis in SD male rats, the following scheme was designed, as shown in Figure 6A. All rats survived well during the treatment and displayed comparable body weight change (Table 1). It was revealed that the CCl_4_ +LDH groups had significantly less change in body weight than the other groups. This could be due to marked hepatic damage. On the other hand, rats that received LDHs-SRF formulations showed a significantly increase in body weight throughout the treatment period (*p* < 0.05). This could reflect the reflect in good biocompatibility of LDH nanoparticles as drug carriers and proper doses of therapeutic agents.

**TABLE 1:** Effect of sorafenib and LDHs-SRF on animals’ body weight after IV administration to fibrotic rats 3 times weekly for 5 weeks.

Treatment groups	Body weight gain (g)
Control^a^	85.7 ± 9.6
CCl_4_+LDHs^d^	−20.5 ± 3.4
CCl_4_+sorafenib^c^	38.6 ± 5.8
CCl_4_+LDHs-sorafinib^b^	50.7 ± 7.5

The study was done on male SD rats of 8 animals in each group (*n* = 8). Values were expressed as mean ± SD. Means of the groups with different superscript letter are significantly differed (a > b > c > d). (*p* ≤ 0.05) by ANOVA, test using Post-hoc Test (Tukey).

Tissue inflammation and collagen deposition were observed by H&E, sirius red and masson staining. H&E staining results showed that compared with the control group, the hepatic lobule structure of rats in the CCl_4_+LDHs group was destroyed, with extensive necrosis of liver cells, exhibiting ballooning degeneration in some cases, and a large number of inflammatory cells infiltrated in the portal area. Compared with the control group, the hepatic cell necrosis and inflammatory response were improved in SRF group and LDHs-SRF group, but hepatic sinusoids were abnormally dilated with some false lobules. In the latter group, hepatic sinusoids were slightly dilated without false lobules. After sirius red and masson staining, a small number of collagen fibers was visible around the central vein and the portal area of the liver in the control group. In contrast, a large amount of collagen fiber deposition was observed in the liver, mostly in the portal area in the CCl_4_+LDHs group. Besides, a small amount of collagen fiber hyperplasia was visible in the portal area, and the hepatic sinusoids were slightly expanded in the SRF group. Finally, collagen fiber deposition was significantly decreased in the LDHs-SRF group (Figure 6B).

Previous *in vitro* studies found that LDHs-SRF significantly inhibited the proliferation and activation of HSC-T6 cells, and down-regulated the expression of activation marker α-SMA in HSC-T6 cells (Supplementary Figure S4). In order to further understand the mechanism of LDHs-SRF *in vivo*, the expressions of α-SMA in liver were detected by immunohistochemistry, and the results showed that the expressions of α-SMA in liver of CCl_4_+LDHs group were significantly increased. LDHs-SRF treatment significantly inhibited the protein expressions of α-SMA in the liver induced by CCl_4_, suggesting that LDHs-SRF could significantly inhibit the continuous activation of CCl_4_-induced liver HSCs cells (Figure 5C).

## Discussion

It is widely acknowledged that hepatic stellate cells are important players in the progression of liver fibrosis. When the liver is injured, it secretes various pro-fibrosis factors, among which TGF-β1 is the most important (Hu et al., 2018). TGF-β1 activates resting hepatic stellate cells by paracrine and autocrine action, leading to an increase in α-SMA, a marker of HSC activation. The continuous activation of HSCs leads to the accumulation of ECM proteins, including collagen (mainly type I, III, and IV) and non-collagen ones (Karsdal et al., 2020). Studies have corroborated that HSCs are the main source of ECM proteins (Shi et al., 2020). In addition, HSCs can be induced to transdifferentiate into myofibroblasts (Zhang et al., 2016). Accordingly, targeting HSCs has become a hot topic in the study of liver fibrosis.

In this study, we confirmed that SRF-loaded LDHs yielded a better therapeutic effect than SRF alone, providing an effective approach for treating liver fibrosis. LDHs-SRF were synthesized by a hydrothermal and co-precipitation method. The successful synthesis of LDHs-SRF was confirmed by XRD, FT-IR, particle size distribution, zeta potential and other characterization methods. The characteristics of LDHs-SRF include a dense particle size distribution and positive charge, conducive to better delivery of SRF to target cells. As shown in Supplementary Figure S1, the significant increase of the average particle size is mainly attributed to rapid protein adsorption on the LDH surface and formation of LDH aggregates. Previous research (Li et al., 2021) have reported that BSA coating on LDH nanoparticles could stabilize the colloidal stability, prolong the blood circulation time, and reduce the nonspecific cellular delivery in physiological environments (e.g., serum, cell culture medium or phosphate buffer saline). It would provide a promising strategy to reduce protein corona formation. Therefore, we will try to construct an BSA stabilized layered double hydroxide nanoparticle (B-LDH) system to load and deliver sorafenib in our future research.

After HSC-T6 cells were treated with 10 μM SRF and LDHs-SRF for 24 h, LDHs-SRF exhibited stronger proliferative inhibition than SRF. Cell apoptosis and cell cycle assays also showed that LDHs-SRF could increase the apoptosis rate of HSC-T6 cells and inhibit cell proliferation, especially HSC-T6 cells in the G2/M phase. It has been established that α-SMA can be used to identify whether HSC-T6 cells are activated (Hemmann et al., 2007). In our study, the immunofluorescence and Western blot assays confirmed that α-SMA expression in HSC-T6 cells was downregulated in both SRF and LDHs-SRF treatment groups, with more significant effects visible in the latter group.

Sorafenib is the earliest targeted drug approved by the FDA to treat advanced liver cancer and is a multi-target receptor tyrosine kinase inhibitor, which targets to inhibit serine/threonine kinases and other signaling pathways, inhibits the proliferation of a variety of tumor cells and promotes apoptosis. During the process of liver fibrosis, the TGF-β1/Smad signaling pathway plays an important role. TGF-β1 is the most important fibrogenic factor, activated TGF-β1 through binding and activating cell surface with serine/threonine II and I TGF-β1 receptor, the formation of protein complex to further activate Smad2 and Smad3 (Liu et al., 2003). Phosphorylated Smad2/3 binds to Smad4 and polymerizes into a complex, which enters the nucleus and binds to DNA connexin to initiate transcription of target genes (Schnabl et al., 2001). In this signaling pathway, Smad7 is an inhibitory Smad (Bian et al., 2014), a negative feedback regulator in the TGF-β signaling pathway that can attenuate TGF-β1 induced HSCs activation and improve liver fibrosis. PI3K/AKT pathway is a classical signaling pathway that has been shown to be involved in various physiological and pathological processes such as cell proliferation and differentiation, apoptosis and growth (Chen et al., 2016). AKT is a serine/threonine-protein kinase, also known as protein kinase B, located downstream of the PI3K/AKT signaling pathway. It activates serine/threonine proteins through substrate phosphorylation, thereby promoting proliferation and anti-apoptosis. Intriguingly, studies found that after suppressing the PI3K/AKT signaling pathway and HSCs proliferation and activation, apoptotic activity was increased, with downregulated expression of some fibrosis-related genes and collagen fibers (Lei et al., 2019; Wang et al., 2019). In addition, other studies have reported that the p-AKT pathway can reduce the activation of HSCs induced by TGF-β1, inhibit their migration and contraction, and reduce collagen I production (Kurniawan et al., 2020). This finding suggests that suppression of the PI3K/AKT signaling pathway could prevent and improve the progression of liver fibrosis (Ji et al., 2021). Accordingly, the TGF-β1/Smad signaling pathway is considered a potential therapeutic target for liver fibrosis. Moreover, in the present study, after treatment with LDHs-SRF, the expression of TGF-β1 and p-AKT/AKT was downregulated, while the expression of Smad7 was upregulated, which significantly inhibited the TGF-β1/Smad and AKT signaling pathways.

Importantly, we found that LDHs-SRF could inhibit EMT signaling. EMT is a complex and dynamic process, which is not only involved in normal physiological processes such as tissue and organ development but also closely related to pathological processes such as tissue injury repair and regeneration and organ fibrosis (Stone et al., 2016; Zhao et al., 2016; Ma et al., 2018). In recent years, it has been documented that HSC activation can be attenuated by blocking the EMT process (Dai et al., 2015; Masola et al., 2019). In addition, TGF-β1 has been established as a strong inducer of EMT (Stone et al., 2016). Interestingly, the TGF-β1/Smad signaling pathway can reportedly mediate EMT, TGF-β1 activated Snail, Slug, ZEB1, Twist and other transcription factors, and upregulate the expression of interstitial cell markers Vimentin and N-cadherin. Moreover, the expression of epithelial cell marker E-cadherin was downregulated (Chilvery et al., 2020; Guo et al., 2021). Our results from the cell scratch assay and Western Blot are consistent with the existing literature.

However, there are some shortcomings in our study. LDHs-SRF nanoparticles in PBS or serum-containing DMEM solution were not well dispersed. Liver tissue in the CCl_4_ group demonstrated cellular damage and centrilobular necrosis. Compared to the drug treatment with SRF alone the LDHs-SRF attenuated the accumulation of collagen in the liver, which was shown by the decrease in Sirius red positive areas. But the difference is not obvious *in vivo*. Due to poor stability of the LDHs-SRF, the desired therapeutic effect is not achieved *in vivo*. We will try to construct an BSA stabilized layered double hydroxide nanoparticle (B-LDH) system to load and deliver sorafenib in our future research. In terms of clinical application, currently, the model of liver fibrosis caused by cholestasis is most reported, while the model of liver fibrosis induced by carbon tetrachloride, which is common in the laboratory, is used in our study, and its clinical application is limited.

In summary, LDHs-SRF inhibited the proliferation and activation of HSC-T6 cells and reduced cell migration ability and ECM protein deposition through TGF-β1/Smad, AKT signaling pathway and EMT pathway. In addition, we evaluated the therapeutic effect of LDHs-SRF on CCl_4_-induced liver fibrosis in SD rats. After LDHs-SRF treatment, the inflammatory infiltrates, and collagen fiber deposition in the liver were significantly reduced, and the survival environment of the liver fibrosis model rats was improved.

## Conclusion

In the present study, we successfully developed LDHs-SRF formulation to ameliorate anti-hepatic fibrosis. LDHs-SRF showed superior ability in cellular uptake of HSC-T6 cell line *in vitro*. Furthermore, LDHs-SRF could induce cell apoptosis. LDHs-SRF induce their anti-fibrosis effects mainly through the PI3K/AKT signaling pathway and inhibited AKT phosphorylation. *In vivo* data and observations confirmed that LDHs-SRF exert significant anti-liver fibrotic ability. Therefore, this nanomedicine is a potential strategy for treating liver fibrosis.

## Data Availability

The raw data supporting the conclusion of this article will be made available by the authors, without undue reservation.

## Supplementary Material

Supplementary Figure S1
**(A)** The average particle size and PDI of LDHs and LDHs-SRF in serum. **(B)** The zeta potential of LDHs and LDHs-SRF in serum.

Supplementary Figure S2 Cellular uptake of LDH-SRF (stained with FITC) at different time points by FACS analysis.

Supplementary Figure S3 The uptake of LDHs-SRF by HSC-T6 cells was observed in TEM. The scale bar: 5 μm.

Supplementary Figure S4 Immunofluorescence imaging of α-SMA expression level (Scale bar = 100 μm).

## Abbreviations

AKT, protein kinase B; BSA, bovine serum albumin; CCK-8, cell counting kit-8; Cy5, cyanine 5; DMEM, dulbecco’s modified eagle medium; EDTA, ethylene diamine tetraacetic acid; EMT, epithelial-mesenchymal transition; FDA, food and drug administration; FITC, fluorescein isothiocyanate; H&E, hematoxylin and eosin; mTOR, mammalian target of rapamycin; p-AKT, phosphorylated protein kinase B; PI, propidium iodide; PI3K, phosphatidylinositol-3-kinase; ROS, reactive oxygen species; SDS-PAGE, sodium dodecyl sulfate polyacrylamide gel electrophoresis; TGF-β1, transforming growth factor-β1; UV-VIS, ultraviolet visible; XRD, x-ray diffraction; ZEB1, zinc finger E-box binding homeobox 1.
