# Supplementary figures and images for: Layered Double Hydroxides-Loaded Sorafenib Inhibit Hepatic Stellate Cells Proliferation and Activation In Vitro and Reduce Fibrosis In Vivo
Source: Front Bioeng Biotechnol. 2022 May 27;10:873971. doi: 10.3389/fbioe.2022.873971 (PMC9196193; doi:10.3389/fbioe.2022.873971)

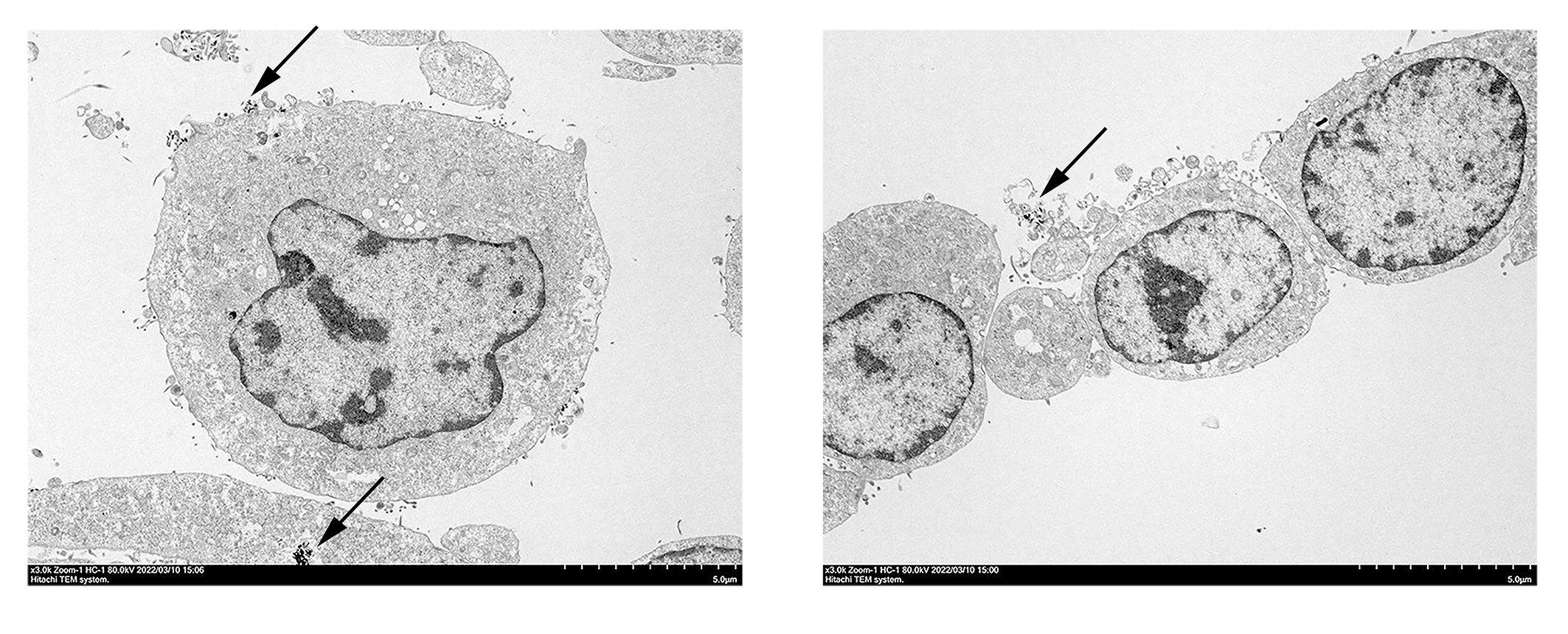

Supplement: Supplementary file 1 [file Image3.TIF]

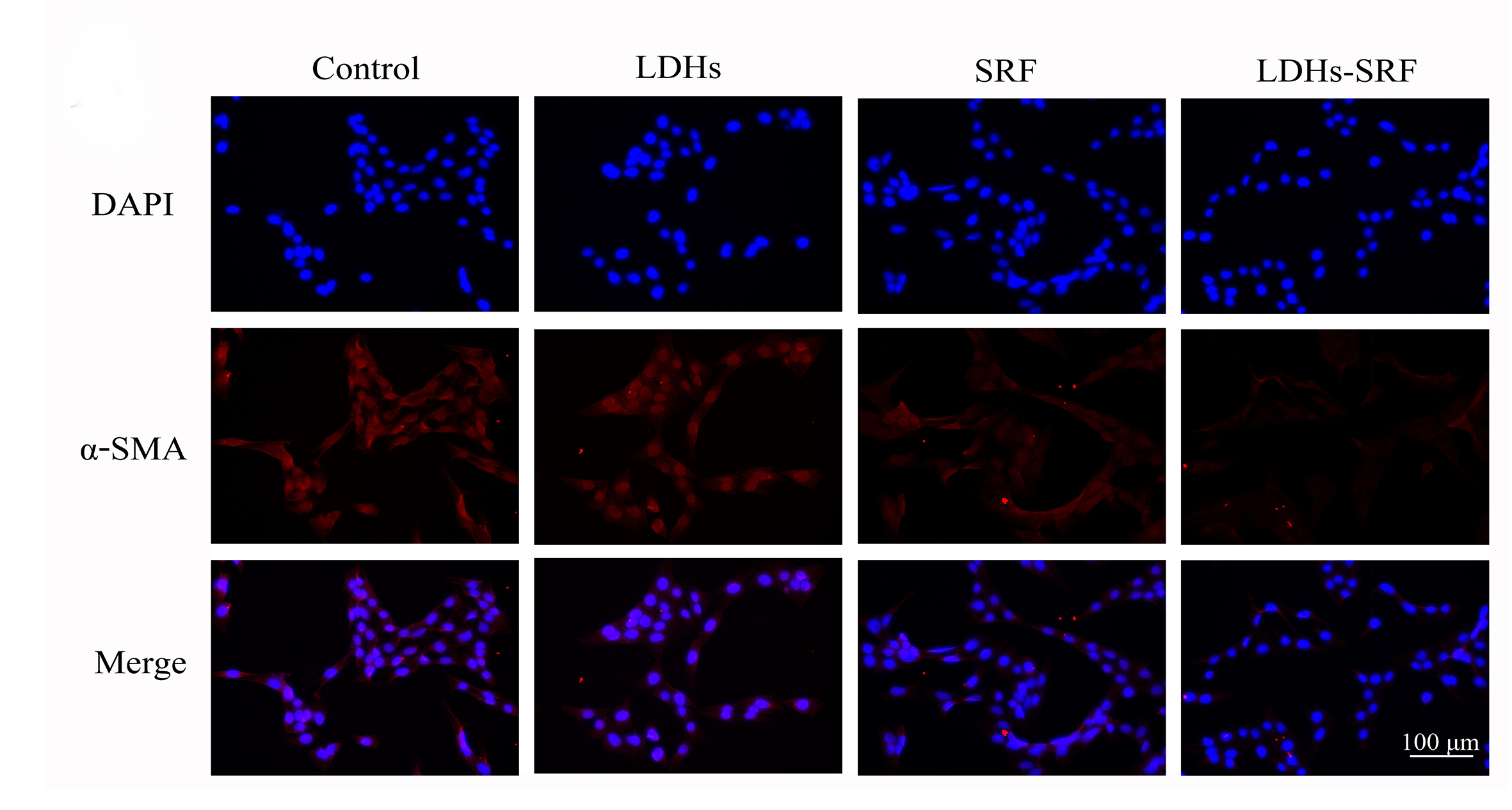

Supplement: Supplementary file 2 [file Image4.TIF]

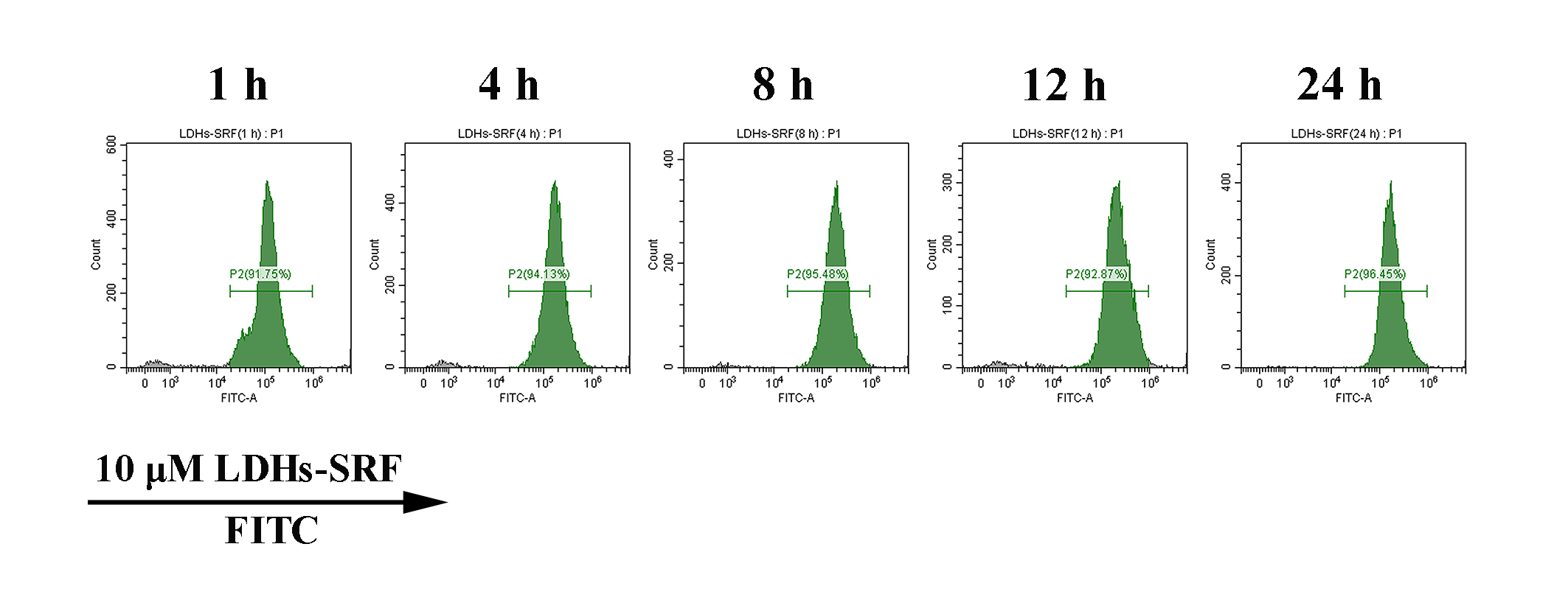

Supplement: Supplementary file 3 [file Image2.TIF]

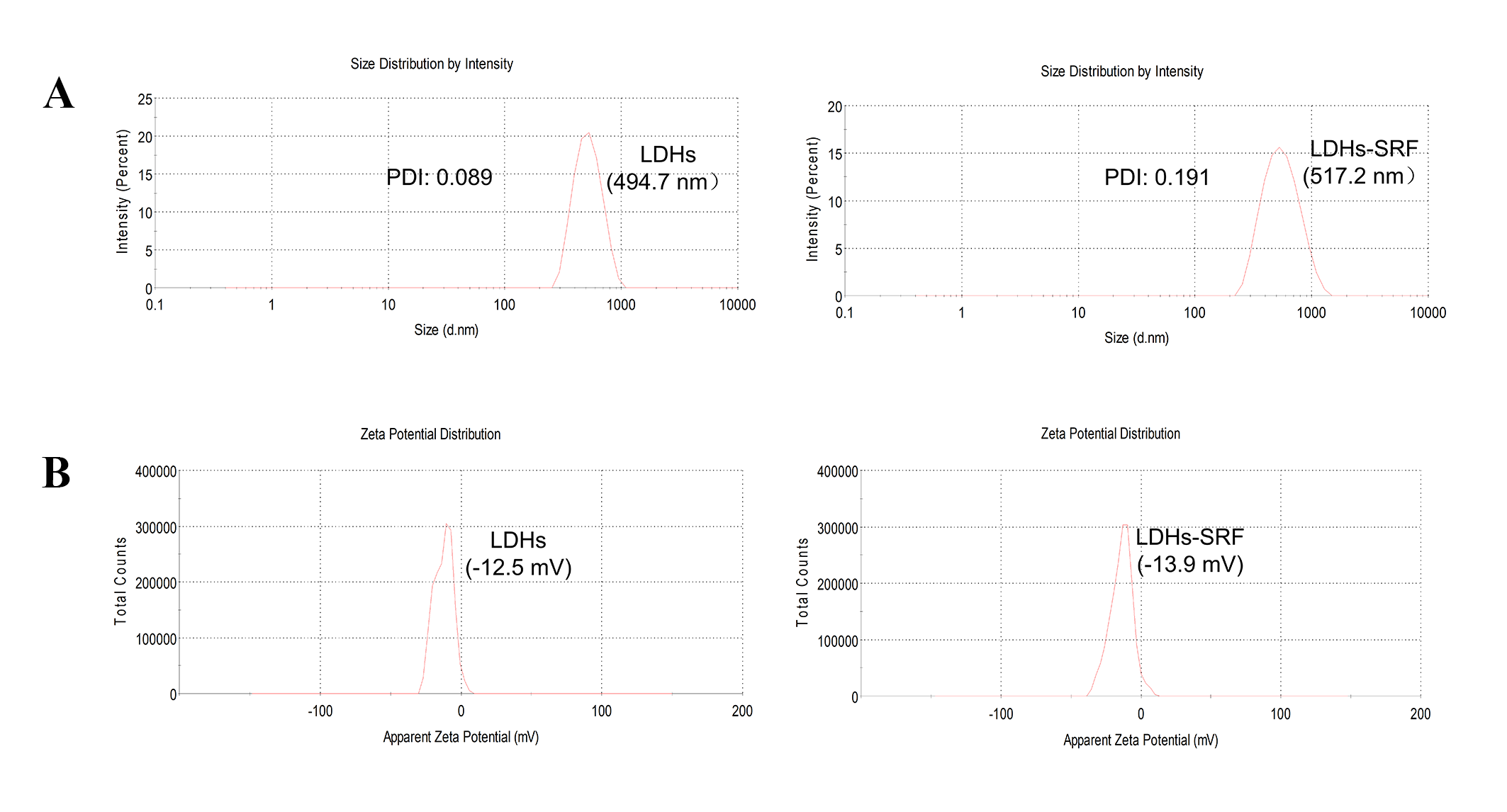

Supplement: Supplementary file 4 [file Image1.TIF]
